# Biological control of *Magnaporthe oryzae* using natively isolated *Bacillus subtilis* G5 from *Oryza officinalis* roots

**DOI:** 10.3389/fmicb.2023.1264000

**Published:** 2023-10-09

**Authors:** Ling-Yun Lei, Zi-Xuan Xiong, Jin-Lu Li, De-Zheng Yang, Liu Li, Ling Chen, Qiao-Fang Zhong, Fu-You Yin, Rong-Xin Li, Zai-Quan Cheng, Su-Qin Xiao

**Affiliations:** ^1^Biotechnology and Germplasm Resources Institute, Yunnan Academy of Agricultural Sciences, Kunming, China; ^2^School of Agriculture, Yunnan University, Kunming, China

**Keywords:** endophytic bacteria, rice blast, *Bacillus subtilis*, disease resistance, genome

## Abstract

Rice blast, caused by *Magnaporthe oryzae*, is a major threat to global rice production causing significant crop losses and impacting grain quality. The annual loss of rice production due to this disease ranges from 10% to 30%. The use of biologically controlled strains, instead of chemical pesticides, to control plant diseases has become a research hotspot. In this study, an antagonistic endophytic bacterial strain was isolated from the roots of *Oryza officinalis* using the traditional isolation and culture methods. A phylogenetic tree based on 16S RNA and whole-genome sequencing identified isolate G5 as a strain of *Bacillus subtilis*. This isolate displayed strong antagonistic effects against different physiological strains of *M. oryzae*. After co-culture in LB medium for 7 days, the inhibition rates of the mycelial growth of four strains of *M. oryzae*, ZB15, WH97, Guy11, and T-39800E were 98.07 ± 0.0034%, 98.59 ± 0.0051%, 99.16 ± 0.0012%, and 98.69 ± 0.0065%, respectively. Isolate G5 significantly inhibited the formation of conidia of *M. oryzae*, with an inhibition rate of 97% at an OD_600_ of 2. Isolate G5 was able to provide 66.81% protection against rice blast under potted conditions. Whole-genome sequencing revealed that the genome size of isolate G5 was 4,065,878 bp, including 4,182 coding genes. Using the anti-SMASH software, 14 secondary metabolite synthesis gene clusters were predicted to encode antifungal substances, such as fengycin, surfactin, and bacilysin. The G5 isolate also contained genes related to plant growth promotion. These findings provide a theoretical basis for expounding the biocontrol mechanisms of this strain and suggest further development of biogenic agents that could effectively inhibit rice blast pathogen growth and reduce crop damage, while being environmentally friendly, conducive to ecological development, and a sustainable alternative to chemical pesticides. This study also enriches the relevant research on endophytes of wild rice, which proves that wild rice is a valuable microbial resource bank.

## 1. Introduction

There are 2.5 to 3.5 billion people around the world who depend on rice (*Oryza sativa* L.) for nutrition; its demand is still growing in some low-income regions (Asibi et al., 2019). As a result of the ascomycete fungus *Magnaporthe oryzae*, rice blast is one of the most devastating rice diseases in the world (Rossman et al., 1990; Komatsu et al., 2016). Rice blast causes an average yield loss of 10% to 30% per year, which represents a global yield loss of approximately 157 million tons annually. Several outbreaks have been reported. An estimated 800,000 tons of rice were lost in Japan during the rice blast epidemic in 1953 (Wang et al., 2014). Rice blast diseases result in the loss of approximately 564,000 tons of rice each year in eastern India alone (Devanna et al., 2022).

Rice blast is a non-organ selective disease. It is possible for *M. oryzae* to infect rice plants at any developmental stage, including the leaves, stems, nodes, panicles, and roots, reducing market value, and affecting food security (Li et al., 2007). Currently, the most important measures for controlling rice blast in rice production are planting disease-resistant varieties and controlling the use of chemical pesticides. These measures have been effective in reducing the impact of rice blast on crop yield; however, they also have limitations. Owing to the high number of different strains and rapid evolution leading to variation in *M. oryzae*, the general disease-resistant varieties lose their resistance after 3–5 years (Dean et al., 2005). The extensive use of chemicals not only increases the drug resistance of pathogenic bacteria and accelerates their mutation but also leads to environmental pollution (Karthikeyan and Gnanamanickam, 2008). A biocontrol agent is increasingly popular because of the fact that it is eco-friendly, has a low residual effect, is highly selectable, and can control an organism for an extended period of time. Therefore, it is important to implement integrated disease management strategies that reasonably apply the necessary agricultural, biological, physical, chemical, and other comprehensive technical measures to economically, safely, and effectively eliminate or control diseases and increase production and income (Ons et al., 2020). Compared with traditional control methods, integrated control can better realize the benign, healthy, and sustainable development of the agricultural economy.

Plant endophytes, including fungi, bacteria, and actinomycetes, which colonize plant organs, tissues, and intercellular spaces, have a stable living space inside the healthy tissues of the host plant (Sturz and Nowak, 2000). These are critical microbial resources whose urgent development has been proven beneficial (Tan and Zou, 2001). Bacteria are dominant components of the plant microbiome and are important endophyte components (Afzal et al., 2019). The probiotic effects of endophytic bacteria on plants include (1) promoting plant nutrient absorption, such as nitrogen, phosphorus, and ions; (2) regulating plant growth and development by regulating plant hormones (auxin, cytokinin, ethylene, etc.) (Davison, 1988); and (3) helping plants resist stresses, including biotic and abiotic stresses.

Endophytic bacteria have direct probiotic effects on plants such as promoting plant nutrient absorption and regulating plant growth and development (Bevivino et al., 1998). Endophytic bacteria indirectly mediate resistance to pathogenic bacteria through a series of biochemical pathways. These biochemical pathways include the secretion of antibiotics and cell wall-degrading enzymes, reduction of ethylene levels in plants, induction of systemic resistance in plants, reduction of the concentration of ions needed by pathogens, and synthesis of volatile organic compounds that inhibit pathogenic bacteria (Santoyo et al., 2016; Glick, 2020).

One of the three wild rice species in China, *Oryza officinalis* Wall., grows mostly in hills or woodlands and warm, rainy, humid environments. *O. officinalis* Wall. has strong growth potential; its biomass is approximately 20 times that of cultivated rice (Kiran et al., 2013). It has well-developed vascular bundles, barren tolerance, shading, photooxidation, and a strong ability to absorb mineral elements simultaneously (Cheng et al., 2005). *O. officinalis* Wall. is known to have endured a variety of harsh conditions and natural calamities over a long period of time, making it a valuable genetic resource for rice breeding (Tan et al., 2004; Devanna et al., 2014). It also harbors a special and effective microbial community ecosystem, which is an ideal source of endophytes for the biocontrol of rice diseases (Tian et al., 2023). Moreover, compared with cultivated rice endophytes and rhizosphere bacteria, there are few studies on wild rice.

*Bacillus* spp. are the most widely used biocontrol bacteria in research and production. A broad range of ecological niches are inhabited by bacteria from this group, such as soil, water, and air, as well as on the surfaces and rhizosphere of plants, in the gastrointestinal tract of animals, and in many extreme environments (Wang et al., 2022). *Bacillus* species exhibit a wide array of secondary metabolisms and possess the capacity to synthesize various structurally distinct antagonistic substances, thereby contributing to their extensive bacteriostatic spectrum within the field of biotechnology (Djordje et al., 2018). Another unique feature arises from their spore-forming ability, which enables them to grow under unfavorable environmental conditions, such as high temperature, drought, ultraviolet light, and other stresses (Siddikee et al., 2010). In addition, their reproductive ability and colonization speed are superior to those of other biocontrol bacteria (Dutta et al., 2014). There are many examples of successful biocontrol strains derived from *Bacillus* spp. *B. thuringiensis* is regarded as the most effective bioinsecticide, showing a good biocontrol effect on most insects of the orders Diptera, Lepidoptera, and Coleoptera (Khan et al., 2022). *B. cereus* HS24, *B. tequilensis* GYLH001, and *B. velezensis* ZW-10 show strong antimicrobial effects against *M. oryzae* (Li et al., 2018; Huang et al., 2019; Chen et al., 2020). *B. subtilis* L1-21 performs well as a biocontrol agent for *Botrytis cinerea* after tomato harvests (Bu et al., 2021) and endophytic *B. subtilis* Lu144 effectively reduces the incidence of mulberry bacterial wilt (Ji et al., 2010). Wei et al. (2011) demonstrated the ability of *B. amyloliquefaciens* to reduce *Ralstonia solanacearum* infections in potato plants.

Most of the beneficial bacteria with biocontrol effects have come from cultivated rice or its rhizosphere soil; however, there are few reports of bacteria isolated from wild rice. The objective of this study was to isolate, screen, and identify biocontrol bacteria in wild rice and to conduct a comprehensive investigation of its potential control effects on *M. oryzae*. Seven microbial strains were isolated from healthy root samples of *O. officinalis* Wall. G5 was the only strain that inhibited *M. oryzae* strongly. An analysis of the strain's morphology and its 16S rRNA sequence identified it as *B. subtilis*. The antagonistic effects of G5 on the four different strains of *M. oryzae* were determined using the plate-confrontation method. Both *in vitro* and field trials were conducted to evaluate the biocontrol effects against *M. oryzae*. Furthermore, we explored the inhibitory mechanisms of *B. subtilis* G5 and the host defense responses against *M. oryzae*. Moreover, the genome of this strain was studied, including the genes involved in antifungal production.

## 2. Materials and methods

### 2.1. Strain, media, and cultural conditions

*M. oryzae* strains, WH97 and Guy11, which are widely used in research, ZB15, which is prevalent in southwestern China, and T-39800E, identified in our previous study, were grown on potato dextrose agar plates at 28°C. The isolated endophytic bacteria were inoculated onto Luria–Bertani (LB) agar plates (Sangong Co., Ltd., Shanghai, China) at 28°C.

### 2.2. Isolation and selection of endophytic bacteria against *M. oryzae* from healthy *O. officinalis* wall

Healthy *O. officinalis* Wall. plants were collected from the Biotechnology and Germplasm Resources Institute of the Yunnan Academy of Agricultural Sciences, Yunnan Province, China. Root samples from wild rice plants were thoroughly washed with tap water to remove airborne counterparts and then soaked in 75% (v/v) ethanol for 1 min to kill common bacteria and some fungi. To further eliminate microorganisms on the surface of plant tissues, all samples were soaked in 1% (v/v) NaClO for 5 min, followed by rinsing five times with sterile water under aseptic conditions for the purpose of depleting epiphytic microorganisms. The surface moisture was removed using sterilized filter paper. The dried tissue was, then, placed in a sterilized mortar and ground; 5 ml of sterile water was added to the ground tissue. After serial dilutions of the suspension, 1 ml aliquots were evenly spread on LB agar plates and incubated at 37°C for 2 to 3 days. Single colonies were selected and purified from LB agar plates by repeated streaking.

To determine the potential antagonistic potential of these isolates, a preliminary screening was conducted using the dual-culture method, as previously described (Ferreira et al., 1991). Plates inoculated only with *M. oryzae* Guy11, which is widely used in research, served as controls. The surface area of the plates was measured by taking photographs with a Canon EOS 77D camera (Canon, Tokyo, Japan) after 7 days of incubation at 28°C. To evaluate the inhibitory effect, the percentage of inhibited growth area was calculated using the formula [(Sc – St)/Sc) × 100], where Sc and St represent the growth area of *M. oryzae* on the control and treated plates, respectively. Each isolate was tested three times with three replicates each. After screening with the above method, strain G5 was isolated and stored at −80°C in glycerol (30%, v/v) stock. As previously described, scanning electron microscopy (SEM; Quanta FEG650, FEI, Hillsboro, USA) (De et al., 2008) was used to observe the morphology and ultrastructure of *M. oryzae* in a dual-culture experiment to assess the antifungal activity of G5′.

### 2.3. Identification of strain G5

The morphological characteristics of strain G5 were examined on LB agar. Genomic DNA extraction from strain G5 was performed using the TaKaRa MiniBEST Bacteria Genomic DNA Extraction Kit Ver.3.0, following the guidelines provided by the manufacturer (TaKaRa, Dalian, China), in order to determine the 16S rRNA gene sequence. The primer sequence is 27F:5′-AGAGTTTGATCCTGGCTCAG-3′; 1492R 5′-GGTTACCTTGTTACGACTT-3′. The 30 μl PCR mixture contained 15 μl of 2 × Phanta Max Master Mix, 1 μl of each 10 μM primer, 4 μl of genomic DNA, and 9 μl of double distilled water. The PCR program was as follows: denaturation at 94°C for 3 min, 30 cycles or 94°C for 15 s, 55°C for 15 s, 68°C for 1.5 min, and final extension at 68°C for 10 min. As a result of PCR product sequencing by Tsingke Biotechnology (Kunming, China), the sequences gained access to the GenBank databases for BLAST searches.

### 2.4. Effects of G5 on conidial germination and appressorium formation

To obtain conidial precipitates, 1 ml of Guy11-GFP conidial suspension was centrifuged at 3,000 rpm/min for 3 min in a 1.5-ml centrifuge tube. In the control group, 1 ml of sterile water was added to each centrifuge tube to suspend the conidial precipitates; 1 ml of G5 at different concentrations (OD_600_ = 1, OD_600_ = 1.5, OD_600_ = 2) was added to each centrifuge tube to suspend the conidial precipitates. One droplet (20 μl) of conidial suspension from each treatment group was placed on a hydrophobic microscope cover glass with wet filter paper and incubated at 28°C in darkness. Conidial germination and appressorium formation were observed under the upright fluorescent microscope (Leica DM2500, Wetzlar, Germany) after 16 h of incubation. In each replicate, we measured the percentage of conidial germination and appressorium formation. There were three replicates of each treatment, with more than 50 conidia assessed for each treatment. The experiment was repeated three times.

### 2.5. Biocontrol assays

#### 2.5.1. Antifungal activity on detached rice leaves

The rice leaf experiment included three groups: prevention, treatment, and control. Leaves from Nipponbare plants at the 5-leaf stage, measuring 5 cm and exhibiting normal growth, were immersed in Petri dishes containing a solution of 6-benzylaminopurine (1 mg/ml). The application of three delicate punctures per leaf segment facilitated the penetration of the treatment agents. Three replicates with ten rice plants each were used in each treatment. Prevention group: 5 μl of the cell-free G5 culture filtrate was applied to punctured leaves, and the leaves were incubated at 28°C in the dark. After 24 h, all puncture sites were inoculated with 5 μl droplets of the conidial suspension of *M. oryzae*, Guy11 (1 × 10^5^ spores/ml). Under alternating light and dark conditions, the leaves were incubated at 28°C for 6 days. Treatment group: The same method as the prevention group was used, but leaves were first inoculated with a spore suspension of *M. oryzae*, and then, the cell-free G5 culture filtrate was added. Control group: Sterile water was used instead of the cell-free G5 culture filtrate; all other steps were the same. After 6 days of incubation, the lesion diameters were compared.

#### 2.5.2. Antifungal activity in greenhouse

The biocontrol effect of *B. subtilis* G5 on rice blast was studied using leaf-spraying inoculation with Nipponbare plants. Each treatment consisted of three replicates with 10 rice plants per replicate. In the prevention experiment, rice seedlings at the 3-leaf stage were subjected to a treatment involving the application of 50 ml of the cell-free G5 culture filtrate. Subsequently, after 7 days, all plants were sprayed with 50 ml conidial suspension of Guy11, containing 1 × 105 spores/ml. The plants were, then, placed in a growth chamber with a temperature of 28°C, humidity of 80%, and subjected to a dark period of 24 h, followed by a light/dark cycle of 12 h each. In the control group, the same procedures were followed, with the exception that the leaves were inoculated with 50 ml of distilled water, followed by the application of 50 ml of the Guy11 conidial suspension (1 × 10^5^ spores/ml).

The disease index was assessed 7 days after spray inoculation. We calculated disease index and biocontrol efficacy as follows: Disease index = [Σ (the number of diseased plants in each disease rating × the number of plants at the corresponding rating)/(total number of plants investigated × the highest disease rating)] × 100%.

Biocontrol efficacy = [(relative disease index of control treatment – disease index of treatment)/disease index of control] × 100%.

### 2.6. Whole-genome sequencing of G5

A NucleoBond^®^ HMW DNA kit (740160.20; Macherey-Nagel, Düren, Germany) was used for high-quality genome extraction of samples according to the manufacturer's instructions. DNA concentration and purity were determined *via* Qubit4.0 (Q33226; Thermo Fisher Scientific, Waltham, MA, USA) and Nanodrop (SMA4000; Taiwan, China). DNA integrity was assessed using 0.75% agarose gel electrophoresis. gDNA was separated into two samples. One sample was randomly selected to build a library with an insertion of 300 bp. The library was processed using the NovaSeq 6000 platform (Illumina, San Diego, CA, USA) and the paired-end 150 bp sequencing strategy, which allowed for the determination of diversity and detection of minor variants, but has the disadvantage of short reads that do not permit the reconstruction of complete haplotypes. The other sample was subjected to end-repair, 3′ adenylated, adapters, and motor protein ligations. The product was purified using Agencourt AMPure XP Beads (A63881; Beckman Coulter, Brea, CA, USA). Finally, fragments larger than 1 kb were screened using single-molecule nanopore DNA sequencing on a MinION Flow Cell (R9.4.1; Oxford Nanopore Technologies, Oxford, UK). Reads longer than the Illumina raw reads were filtered and assembled using Canu software version 1.3 with the default parameters. The genomic sequences were proofread using NextPolish (v1.4.1) and Pilon (v1.18). The assembled sequences were deposited in the NCBI (BioProject ID: PRJNA997266). Gene prediction and annotation were performed using Prokka (version 1.10) and the National Center for Biotechnology Information NR database. Functional annotation was performed on protein-coding genes using the cluster of NCBI non-redundant (NR) protein sequences, Swiss-Prot, Orthologous Groups of Proteins (COG), and Kyoto Encyclopedia of Genes and Genomes (KEGG) databases. Secondary metabolite gene clusters were predicted using antiSMASH v4.1.0 as previously described (Kai et al., 2017). Sequencing was performed by Sangon Biotech Co. Ltd. (Shanghai, China).

### 2.7. Statistical analysis

Statistical analyses were performed using SPSS software version 26 (SPSS, Chicago, IL, United States). All treatments were performed in triplicate. Data are expressed as the mean ± standard deviation (SD). The means of the different treatment groups were determined using one-way ANOVA with Duncan's multiple range test to determine whether there were significant differences between the treatment groups. A p-value of < 0.05 was considered to be statistically significant.

## 3. Results

### 3.1. Isolation of endophytic actinobacterial antagonists against *M. oryzae*

**Figure 1:**
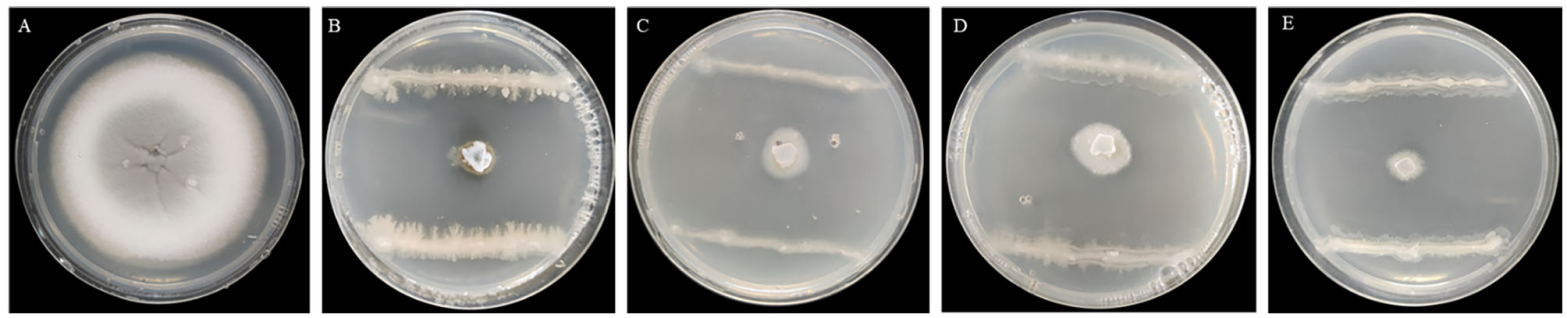
Inhibition effect of G5 on mycelial growth of *M. oryzae* of different physiological races Guy11 **(B)**, WH97 **(C)**, ZB15 **(D)**, T-39800E **(E)**, and CK **(A)**.

Seven endophytic strains were isolated from the roots of healthy *O. officinalis* plants. Among them, one strain, named G5, showed the strongest antagonistic activity against *M. oryzae* Guy11 (Figures 1A, B). Strain G5 showed strong inhibitory activity against all tested strains: WH97, Guy11, ZB15, and T-39800E. The highest percentage inhibition (99.16 ± 0.0012%) was observed for G5 against Guy11, and the lowest recorded (98.07 ± 0.0034%) was observed against ZB15 (Figure 1D). G5 also had an inhibitory effect on WH97 (Figure 1C) and T-39800E (Figure 1E), with inhibition rates of 98.59 ± 0.0051% and 98.69 ± 0.0065%, respectively (Table 1). These results suggest that strain G5 has a strong inhibitory effect on different strains of *M. oryzae*, although the inhibition rate of the G5 strain against different strains is slightly different.

**Table 1:** Inhibition effect of G5 on mycelial growth of *M. oryzae* of different physiological races.

**Physiological races**	**Inhibition rate (%) ± SD**
Guy11	99.16 ± 0.0012 b
WH97	98.59 ± 0.0051 ab
ZB15	98.07 ± 0.0034 a
T-39800E	98.69 ± 0.0065 ab

### 3.2. Identification of endophytic bacterium G5

**Figure 2:**
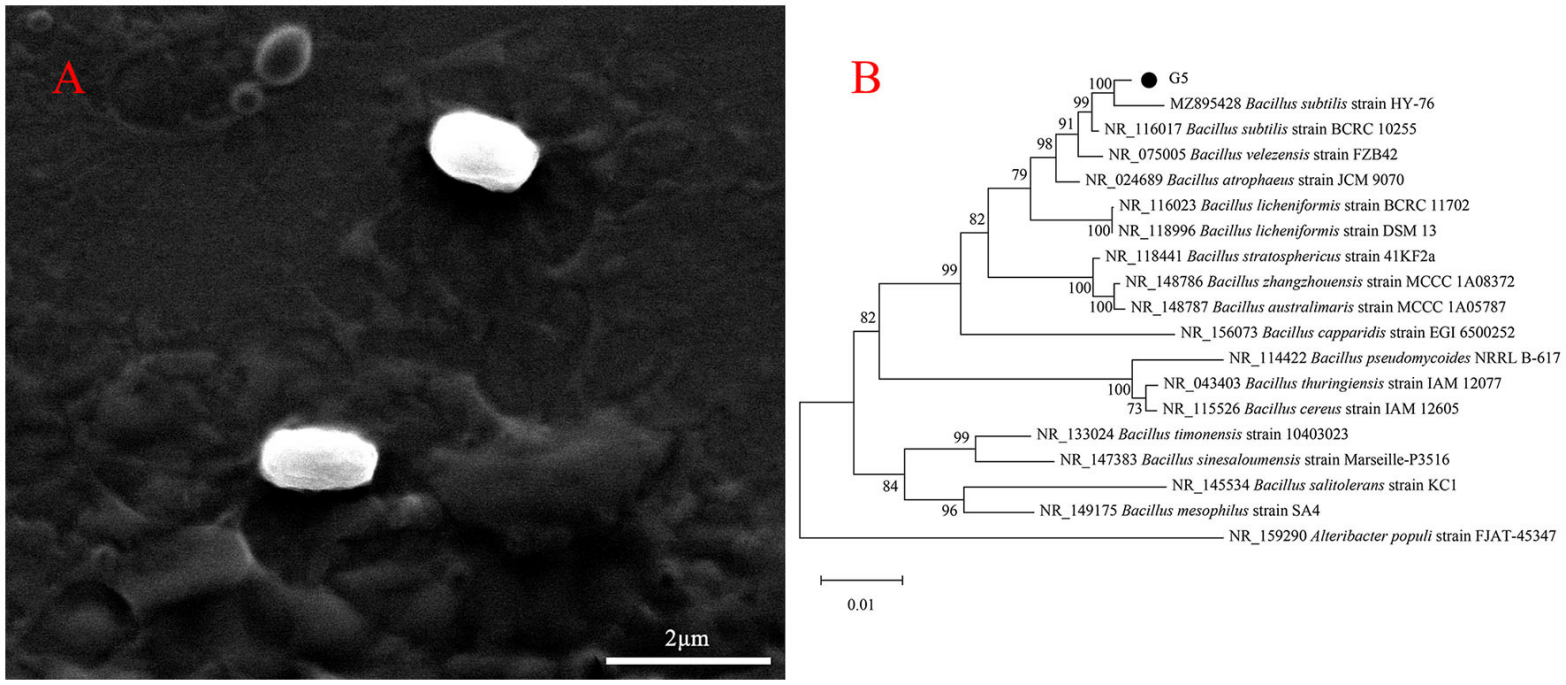
Morphological characteristics of G5 were visualized by SEM **(A)**. Neighbor-joining phylogenetic tree showing the position of *B. subtilis* G5 isolate with other species of *Bacillus* spp. and related taxa based on 16S rDNA gene sequences. Bootstrap values (expressed as percentages of 1,000 replications) are indicated at tree branch points **(B)**.

The colony characteristics of bacteria cultured under certain conditions, including surface, size, color, edge, texture, and shape, have a certain stability, which is important for identifying the types of bacteria. Gram staining is a common method for observing the morphology of bacteria, the results of which can be used to initially classify bacteria. Therefore, preliminary morphological observations were performed on strain G5. On LB medium, G5 colonies showed typical characteristics of *Bacillus sp*., such as being dry and round with irregular protrusions near the margin (Supplementary Figure 1). Based on SEM images, the cells were short rod-shaped structures approximately 0.63–0.72 μm in width and 1.40–1.46 μm in length (Figure 2A). Gram staining of these cells was positive (Supplementary Figure 1). In addition, BLAST analysis of the 16s rRNA genes showed that G5 (GenBank database accession number: OQ874691) shared 99% identity with *Bacillus subtilis* (MZ895428.1 and MZ895403.1). Based on phylogenetic analysis, strain G5 clustered closely with *Bacillus* spp. Furthermore, strain G5 and *B. subtilis* clustered together (Figure 2B).

### 3.3. The morphological and ultrastructural changes in m. oryzae after confrontation with G5

**Figure 3:**
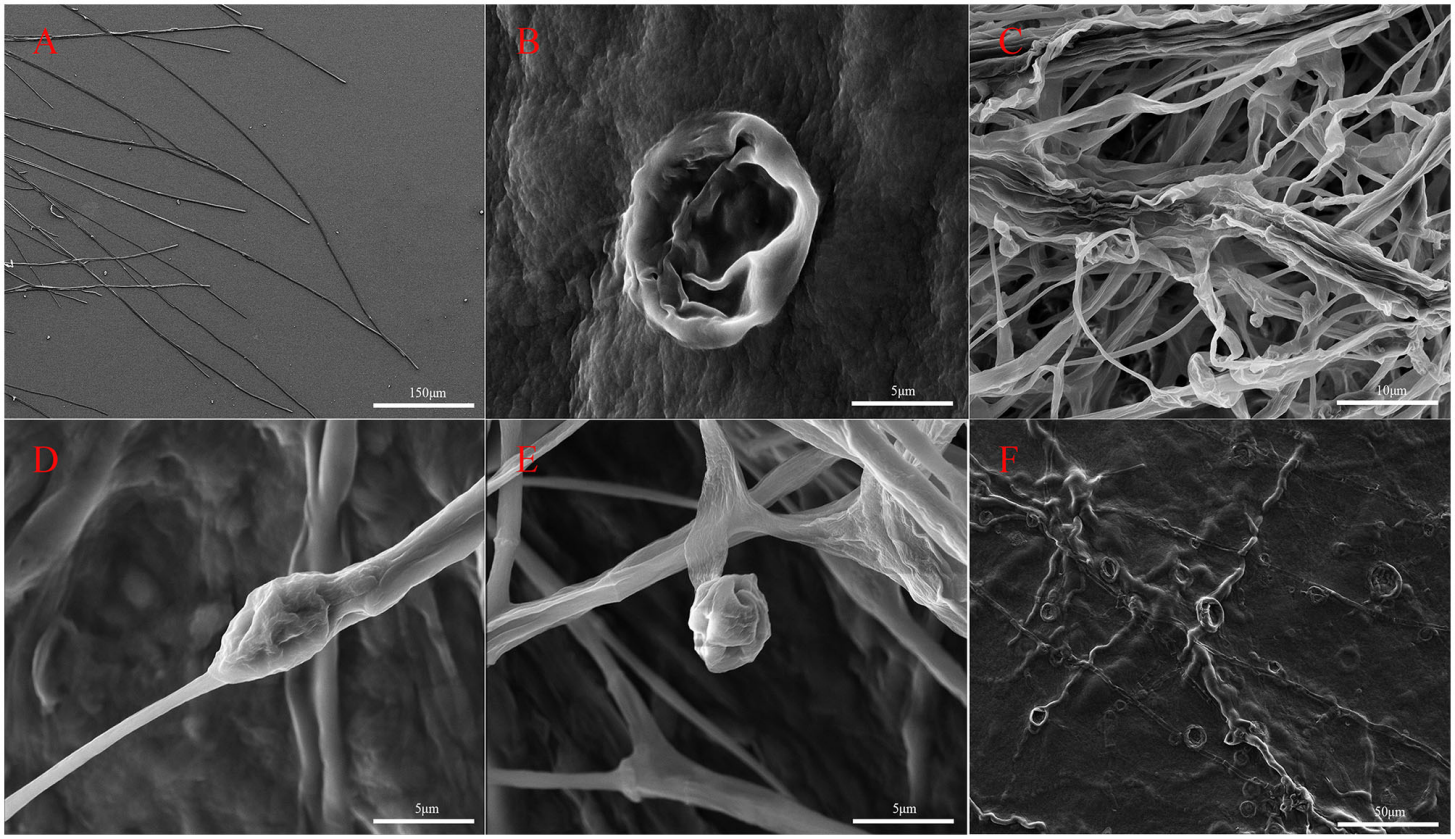
Scanning electron micrographs of *M. oryzae* mycelia control group **(A)** and treated with strain G5 (remaining pictures) after 7 days. *M. oryzae* mycelia treated with strain G5 were wrinkled **(B)**, twisted **(E, F)**, and locally inflated **(C)**, and hyphal tips were deformed **(D)**.

Hyphal structures of *M. oryzae* were observed using SEM (Figure 3). It was observed that the untreated hyphae of *M. oryzae* grew normally, showed a straight, uniform appearance, and had a well-developed tube-like structure (Figure 3A). Conversely, abnormalities were observed in fungal hyphae co-cultured with G5 including deformities (Figure 3B), irregular distortions (Figures 3E, F), and inflated (Figure 3D) and shrunken structures (Figure 3C). These phenomena suggest that some substances produced by strain G5 can inhibit the growth of mycelia and destroy the mycelial cell membrane, resulting in mycelial shrinkage.

### 3.4. Effects of G5 on conidial germination and appressorium formation

**Figure 4:**
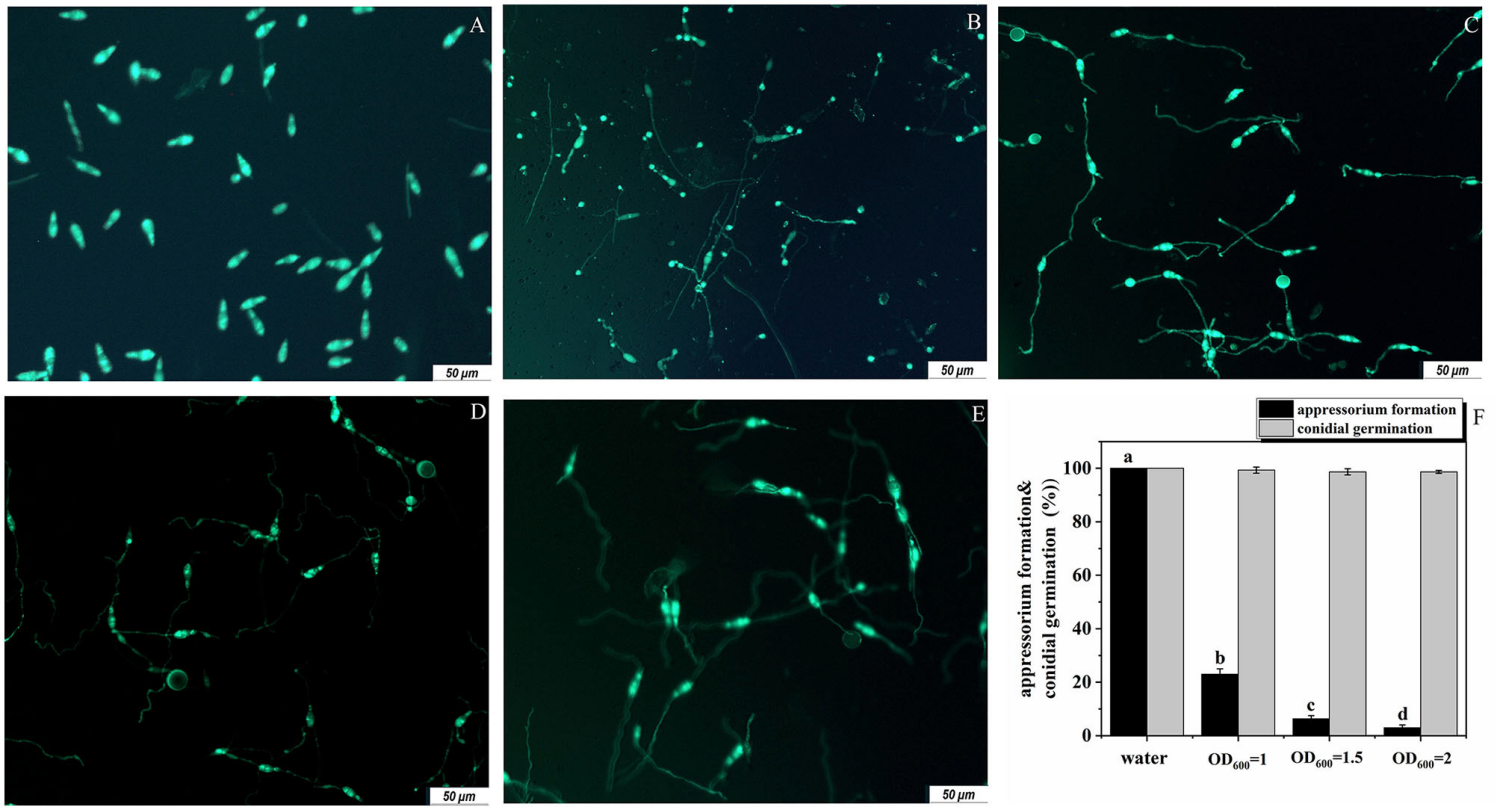
Effect of *B. subtilis* G5 at various concentrations on conidial germination and appressorium formation at 16 hpi. Conidia of strain Guy11-GFP **(A)** were treated with sterile water **(B)** and G5 at the concentrations indicated. Conidium development was observed with sterile water and G5 OD_600_ = 1 **(C)**, OD_600_ = 1.5 **(D)**, and OD_600_ = 2 **(E)**. Conidia examined by differential interference fluorescence microscope. The scale bar indicates 50 μm, conidial germination and appressorium formation with sterile water and G5 at various concentrations. Data from three biological replicates were used to calculate the mean and standard deviation. Mean separation letters indicate statistical significance (*P* < 0.05) based on a one-way analysis of variance followed by the LSD test **(F)**.

For *M. oryzae*, successful infection of rice depends on both conidia germination and the formation of appressoria. Therefore, we studied the antagonistic effects of the strain G5 on these two processes of *M. oryzae*. G5 did not strongly inhibit conidial germination. At 16 hpi (hour post-infection), none of the G5 concentrations inhibited conidial germination. At all concentrations, OD_600_ = 1, OD_600_ = 1.5, and OD_600_ = 2, G5 allowed 99.33%, 98%, and 98.67% of the conidia to germinate, respectively. When the conidia were treated with G5 at OD_600_ = 2 and OD_600_ = 1.5, only 3.00% (Figure 4E) and 6.33% (Figure 4D) of the conidia formed appressoria, respectively. G5 at a lower concentration (OD_600_ = 1) did not completely impair appressorium formation; 23% of the conidia formed appressoria (Figure 4C). These results show that the inhibition of G5 on *M. oryzae* is mainly achieved by inhibiting the formation of the appressorium, with almost no inhibitory effect on conidial germination (Figure 4F). We speculated that G5 interferes with the infection mechanism of *M. oryzae*.

### 3.5. Biocontrol assays

#### 3.5.1. Antifungal activity on detached rice leaves

**Figure 5:**
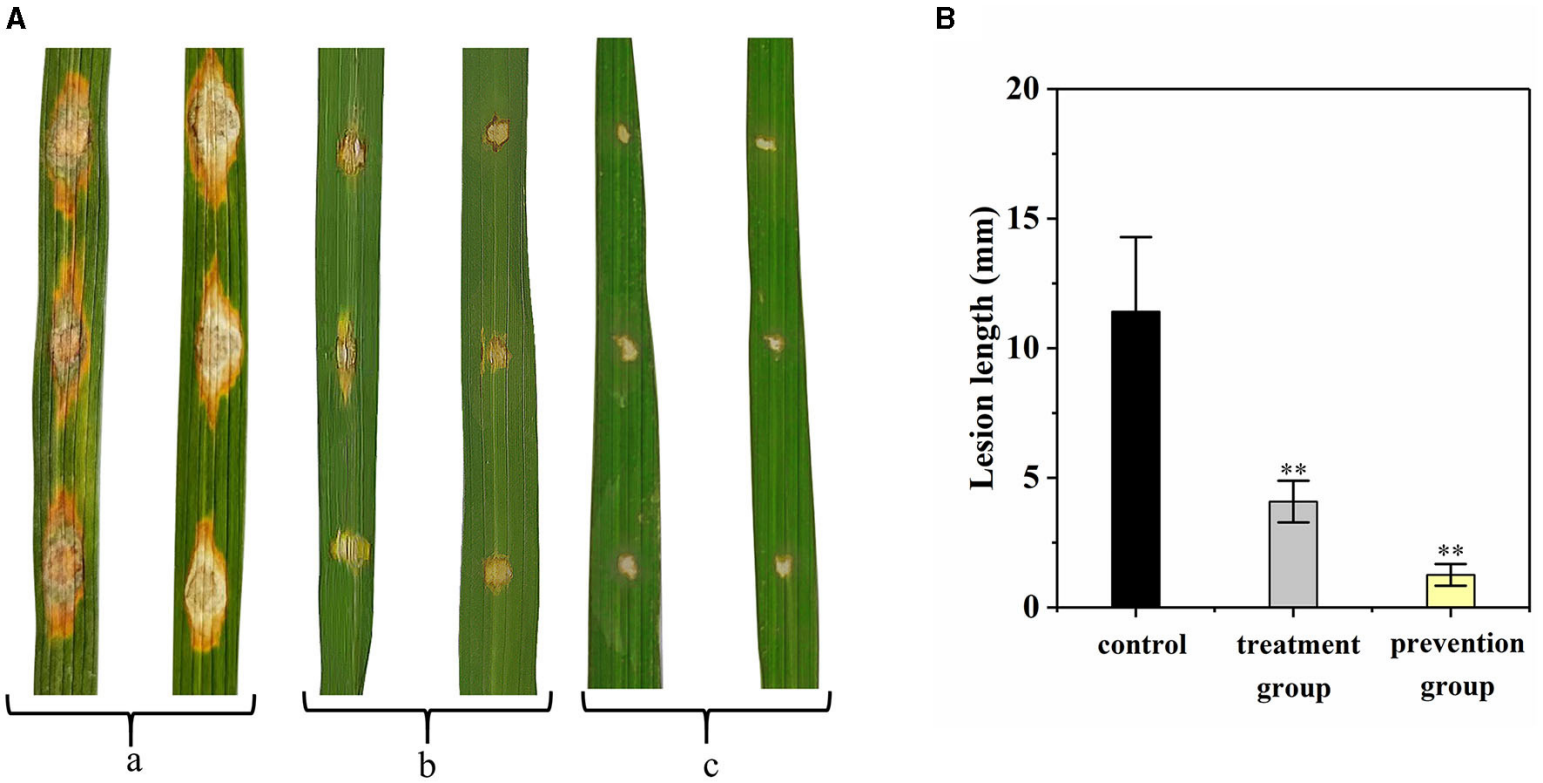
Disease control efficacy on detached rice leaves of G5 against rice blast. (a) Control group. (b) Treatment group. (c) Prevention group. The length of leaf lesions *in vitro* was analyzed **(A)**. Independent samples t-test analyzed data. The symbols ** indicate significant differences at *P* < 0.01 **(B)**.

The disease control efficacy of G5 on the detached rice leaves against rice blast is shown in Figure 5. Compared with the control group (11.42 ± 2.87 mm), the length of leaf lesion of the prevention group was significantly smaller (1.25 ± 0.42 mm; Figure 5A); the length of leaf lesion was also smaller in the treatment group (4.08 ± 0.80 mm). These results indicate that G5 displays a significant preventive effect that was greater than its curative effect (Figure 5B).

#### 3.5.2. Antifungal activity in greenhouse

**Figure 6:**
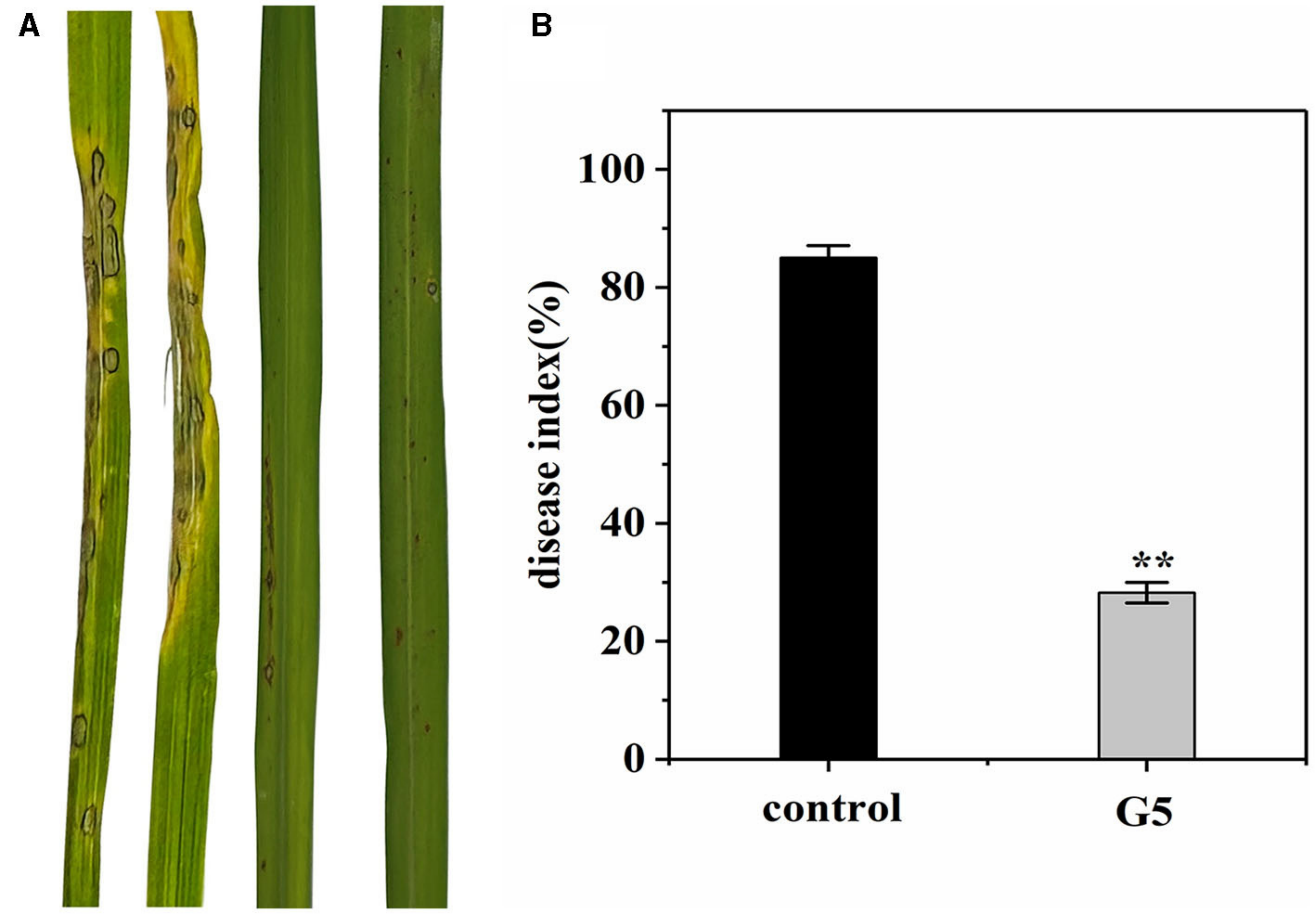
Effect of G5 on resistance against rice blast under greenhouse conditions. The severity of devastating symptoms on the leaves of G5-inoculated rice compared with control under greenhouse conditions **(A)**. According to the disease classification, the disease index of G5-infected and control-infected rice was calculated **(B)**. Bar charts were plotted with mean ± SD from 30 plants, respectively. Independent samples *t*-test analyzed data. The symbols ** indicate significant differences at *P* < 0.01.

We investigated whether G5 confers resistance to rice against rice blast in a greenhouse. Before 7 days of Guy11 inoculation, an inhibitory effect of G5 was observed in rice blast infection. Treating rice plants with G5 strains significantly reduced rice blast. The severity of disease in the control rice plants was observed to be significant, characterized by the formation of large circular or oval brown spots and dense disease spots. In comparison with the disease index of 85.04% observed in rice treated with sterile water, the disease index of rice treated with G5 was significantly lower at 28.23%, indicating a notable biocontrol effect of 66.81% (Figures 6A, B). Additionally, the treatment group exhibited a reduced leaf area covered by diseased spots, accompanied by the limited presence of necrotic spots. In conclusion, G5 has a positive control effect on rice blast.

### 3.6. Genome sequence assembly and general features of G5

**Figure 7:**
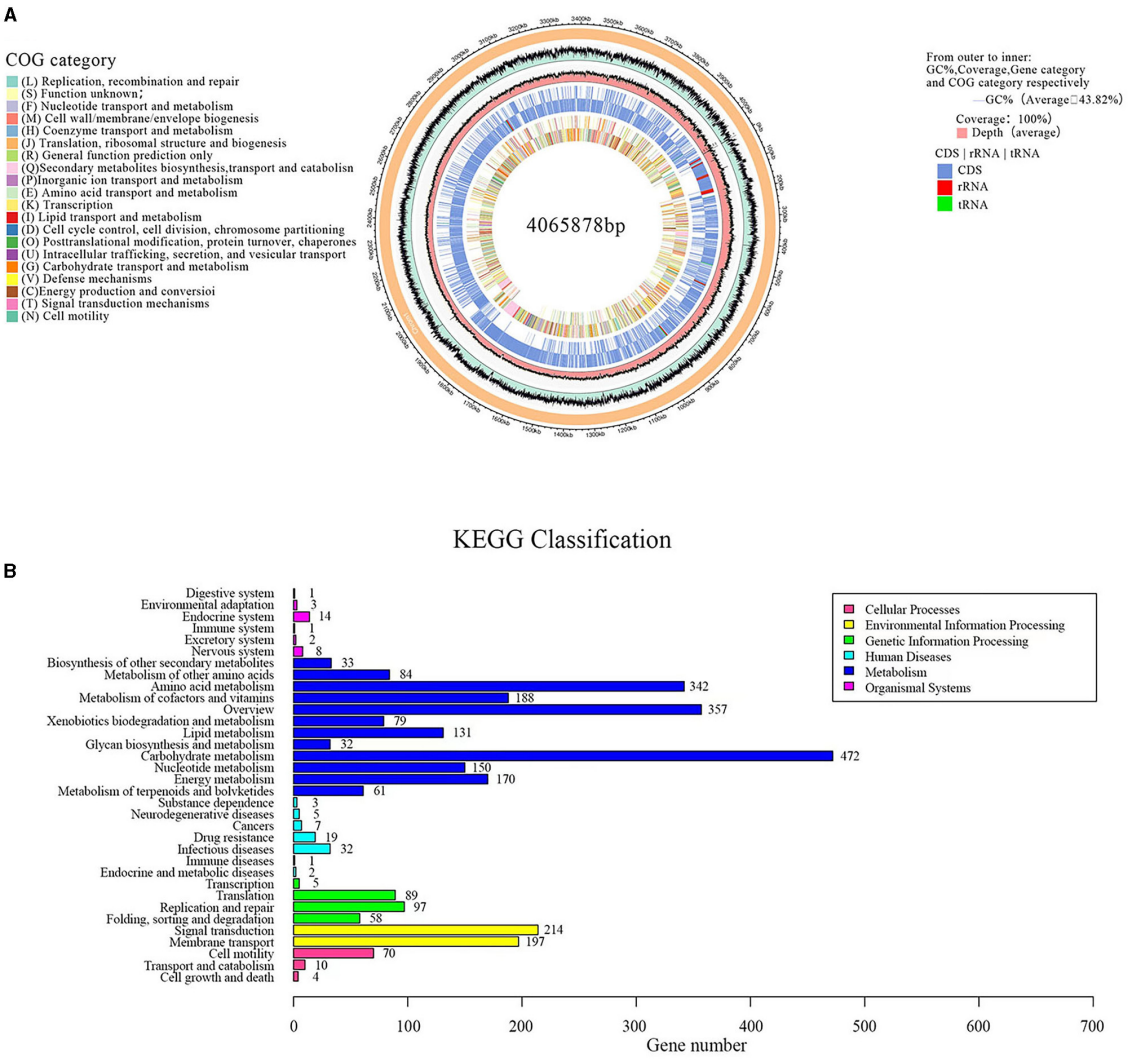
Circular genome map of endophytic *B. subtilis* G5 **(A)**. From outside to inside, circle 1, the size of the complete genome, each scale represents 20 kb; circle 2, G + C content; circle 3, coverage; circles 4 and 5, gene category on the + and – strands, CDS in light blue, tRNA in red, tRNA in green; circles 6 and 7, the predicted protein-coding genes on the + and – strands, different colors represent different COG function classification. KEGG pathway classification **(B)**. The vertical axis is the name of the metabolic pathway involved, and the horizontal axis is the number of genes annotated to that pathway.

Sequencing and analyzing the whole genome of G5 were completed. Accordingly, the G5 genome comprised a singular circular chromosome measuring 4,065,878 base pairs, exhibiting an average GC content of 43.82%. The genome was found to contain 30 rRNA, 86 tRNA, and 29 sRNA genes, as shown in Figure 7A. Furthermore, all 4,182 predicted open reading frames underwent annotation analysis by comparing with the Nr, Swiss-Prot, COG, and KEGG databases, resulting in the annotation of 3,977 candidate genes. Supplementary Figure 2 displays the functional annotations of the genome. COG analysis further categorized 3,015 genes into 26 functional groups, as presented in Figure 7A. The results revealed four main functional gene classes: amino acid transport and metabolism (251 genes), carbohydrate transport and metabolism (258 genes), transcription (256 genes), and cell wall/membrane/envelope biogenesis (181 genes), which represented 31.83% of the genes predicted in the COG analysis. Additionally, other gene clusters associated with inorganic ion transport and metabolism (173 genes), translation, ribosomal structure, and biogenesis (165 genes), energy production and conversion (164 genes), replication, recombination, and repair (108 genes), and coenzyme transport and metabolism (121 genes) accounted for 20.23% of the predicted genes. Moreover, 24.7% of the predicted genes were associated with general function prediction only; unknown functions were poorly characterized. It is worth noting that 54 genes involved in defense mechanisms were annotated, of which 25 encoded the ABC-type multidrug transport system, 10 encoded the resistance protein ABC-type antimicrobial peptide transport system, five encoded the resistance protein beta-lactamase, four encoded the Na^+^-driven multidrug efflux pump, two encoded the multidrug resistance efflux pump, two encoded the microcin C7 resistance protein, one encoded streptogramin lyase, and one encoded the vancomycin resistance protein. These genes may be involved in the antibacterial function of G5 through the synthesis and transport of antibiotics.

A total of 2,250 genes were mapped to five KEGG branches, including cellular processes, environmental information processing, genetic information processing, metabolism, and organismal systems. Among these, a high proportion of the annotated genes was assigned to metabolism, especially the pathways belonging to carbohydrate metabolism (474 genes), overview maps (357 genes), and amino acid metabolism (342 genes) (Figure 7B). Similar to the COG annotations, carbohydrate metabolism was emphasized in the KEGG pathways. In addition, 35 genes were annotated for the biosynthesis of secondary metabolites, such as penicillin and cephalosporin (EC: 3.5.2.6), and 61 genes were annotated for terpenoid and polyketide metabolism, including terpenoid backbone biosynthesis (EC: 2.7.1.148; EC: 2.7.7.60), non-ribosomal peptide structure, and butanoate metabolism (EC: 2.3.3.10; EC: 2.3.1.9). These genes have been speculated to be closely related to the production of G5 antibacterial substances.

### 3.7. Genetic basis for pathogen inhibition

The secondary metabolites of the G5 genome were analyzed using anti-SMASH. Fourteen secondary metabolite gene clusters were predicted (Table 2), including eight highly similar antibiotic synthesis gene clusters: two cyclic lipopeptides (surfactin and fengycin), one polyketide antibiotic (bacillaene), one siderophore (bacillibactin), one dipeptide (bacilysin), one lanthipeptide (subtilin), one thiopeptide (subtilosin A), and one antibiotic gene cluster (thailanstatin A). Fengycin has strong antifungal activity against filamentous fungi; surfactin and bacilysin also exhibit antifungal activities (Ongena and Jacques, 2008). Therefore, the observed inhibitory effect of the G5 strain on *M. oryzae* may have been the result of the combined action of these three metabolites. *B. subtilis* G5 also had four gene clusters with unknown functions, including two terpene clusters, one tRNA-dependent cyclodipeptide synthase cluster, one type III polyketide synthase (PKS) cluster, one lanthipeptide class-I cluster, and one RiPP (ribosomally synthesized and post-translationally modified peptides)-like cluster, suggesting that some *B. subtilis* G5 gene clusters may synthesize new antibacterial substances. The simultaneous production of these antimicrobial metabolites may exhibit synergistic properties in combating pathogens. These metabolites serve as the foundation for pathogen suppression by this endophytic bacterium. Supplementary genes associated with pathogen suppression are presented in Table 2.

**Table 2:** Antimicrobial secondary metabolite clusters in *B. subtilis* G5 genome.

**Region**	**Type**	**Location**	**Most similar known cluster (% of genes show similarity)**	**Effect against**
Region 1	NPRS	349,782–413,221	Surfactin (82%)	Fungi, Bacteria
Region 2	Terpene	1,109,958–1,130,469		
Region 3	lanthipeptide-class-i	1,684,110–1,710,209		
Region 4	NRPS-TransATPKS	1,732,476–1,837,712	Bacillaene (100%)	Bacteria
Region 5	Betalactone	1,910,290–1,987,090	Fengycin (100%)	Fungi
Region 6	Terpene	2,052,891–2,074,789		
Region 7	T3PKS	2,123,258–2,164,355		
Region 8	NRPS	3,092,097–3,139,233	Bacillibactin (100%)	Microbial competitors
Region 9	Lanthipeptide-class-i	3,290,104–3,316,329	Subtilin (100%)	Bacteria
Region 10	CDPS	3,434,015–3,454,761		
Region 11	Sactipeptide	3,679,485–3,701,096	subtilosin A (100%)	Bacteria
Region 12	Epipeptide	3,978,009–3,999,707	thailanstatin A (10%)	human cancer cell lines
Region 13	RiPP-like	3,949,961-3,962,692		
Region 14	other	3,707,880-3,749,298	Bacilysin (100%)	Fungi, Bacteria

### 3.8. Genetic basis for plant growth promotion

In the G5 genome, numerous genes/gene clusters linked to the promotion of plant growth were detected. These encompass genes involved in siderophore synthesis, 3-hydroxy-2-butanone synthesis, and nutrient utilization. Additionally, genes accountable for spermidine synthesis, such as spermidine synthase (*speE*), arginine decarboxylase (*speA*), and agmatinase (*speB*), were identified. Spermidine is a polyamine that promotes plant growth by inducing expansin-related gene expression and inhibiting ethylene synthesis. Siderophores help plants by delivering iron or inhibiting the growth of phytopathogenic fungi by competing for iron (Arguelles-Arias et al., 2009). The genome analysis revealed the presence of genes associated with the synthesis of the volatile bio-promoting metabolite 3-hydroxy-2-butanone, such as acetolactate decarboxylase (alsD), acetolactate synthase large subunit (ilvB), and acetolactate synthase small subunit (ilvH). This metabolite has been shown to enhance plant growth by increasing plant fresh weight. Furthermore, the identification of genes involved in nitrogen and magnesium utilization, such as nitrite reductase large subunit (*nasD*), nitrite reductase small subunit (*nasE*), nitrogen regulatory protein (*nrgB*), ammonium transporter (*nrgA*), nitrate transporter (*nark*), and Mg^2+^ transporter (*mgtE*), was also accomplished. It is worth noting that these genes have the potential to augment nutrient accessibility. Moreover, Table 3 provides a comprehensive summary of additional genes that have been identified in relation to the promotion of plant growth.

**Table 3:** Genes related to plant growth promotion in the G5 genome.

**Gene**	**Gene start**	**Gene end**	**Gene_strand**	**Gene product**	**Function**
*speE*	3705998	3706828	-	Spermidine synthase	Spermidine synthesis
*speA*	37170	38612	+	Arginine decarboxylase	
*speB*	3705065	3705937	-	Agmatinase	
*alsS*	3556477	3556477	-	Acetolactate synthase	3-hydroxy-2-butanone synthesis
*alsD*	3553933	3554700	-	Alpha-acetolactate decarboxylase	
*ilvB*	2726739	2728463	-	Acetolactate synthase large subunit	
*ilvH*	2726224	2726742	-	Acetolactate synthase small subunit	
*nasD*	347244	349661	-	Nitrite reductase large subunit	Nitrogen utilization
*nasE*	346892	347212	-	Nitrite reductase small subunit	
*nrgB*	3611386	3611736	+	Nitrogen regulatory protein	
*nrgA*	3610160	3611374	+	Ammonium transporter	
*nark*	3685754	3686983	-	Nitrate transporter	
*mgtE*	692692	693384	-	Mg^2+^ transporter	Magnesium utilization
*corA*	834831	835790	-	Magnesium transport protein CorA	

## 4. Discussion

Plant roots create a favorable environment for endophytes, offering shelter and nourishment. This enables the endophytes to establish symbiotic connections with host plants. Nutrients discharged through root exudation serve as a continuous source of nutrients for endophytes, supporting essential metabolic functions and overall plant health, even under challenging conditions. Indeed, the pivotal role of endophytic communities in enhancing wild rice adaptability to harsh conditions is well documented (Urs et al., 2013; Zhu et al., 2022). These microorganisms bolster the plant's resilience against diverse stresses, such as drought, salinity, heavy metals, and pathogens.

**Figure 8:**
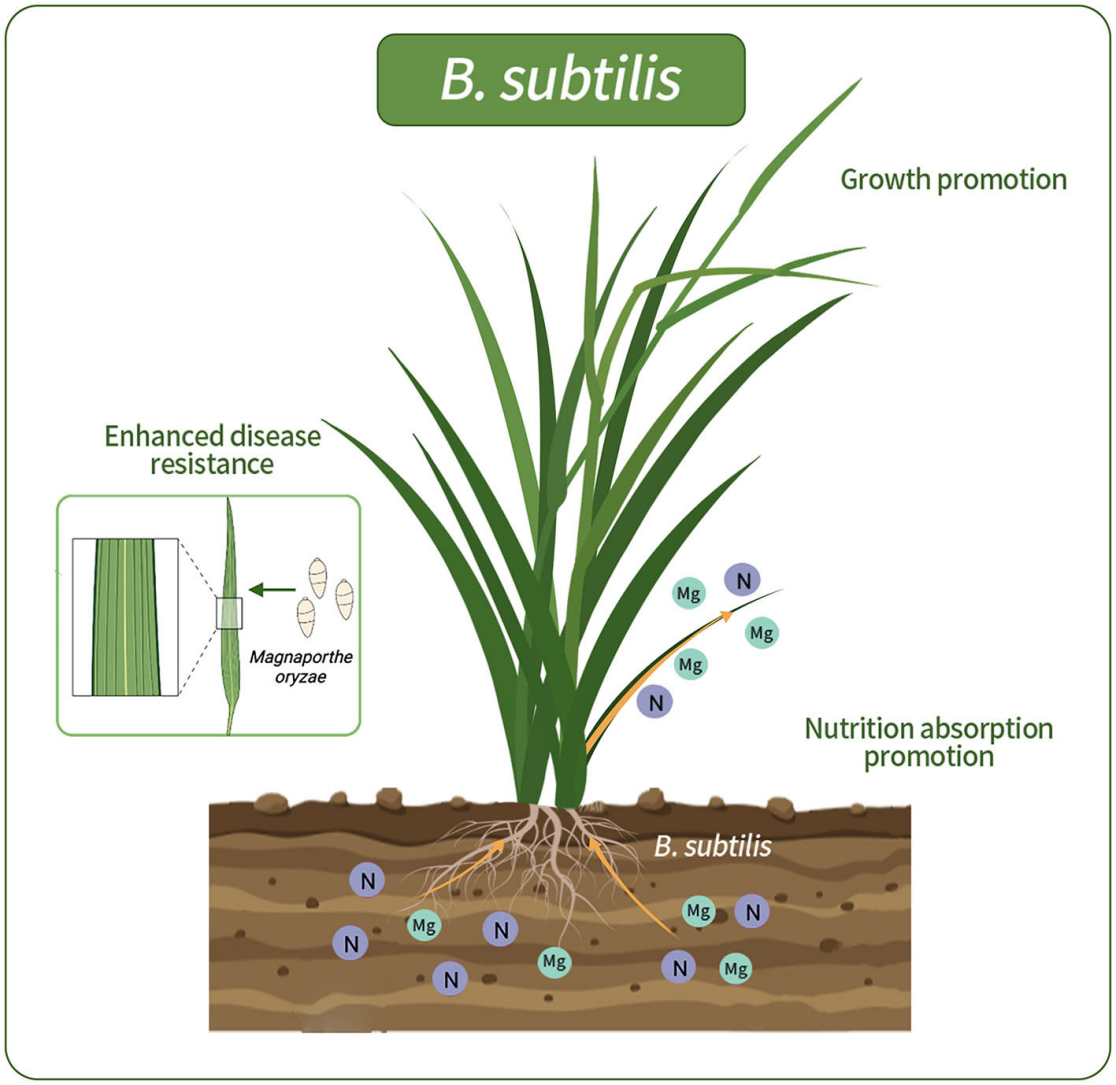
Schematic representations of rice colonized by G5. G5 promoted the absorption of N and Mg elements, promoted rice growth, and enhanced the resistance to rice blast.

In our study, the isolation of *B. subtilis* from wild rice roots revealed its potential to substantially enhance nutrient uptake and root development, thus contributing to the overall plant wellbeing. The incorporation of *B. subtilis* into wild-type rice roots yielded notable benefits. Ameliorating nutrient absorption can significantly enhance growth and concurrently provide biocontrol against rice blast disease. Visual representations elucidate the dynamics of bacterial interactions with host plants (Figure 8). There have also been previous reports on the positive interplay between *B. subtilis* and host plants. *B. subtilis*, a prominent plant growth-promoting rhizobacterium, is an example of this in the field (Earl et al., 2008; Todorova and Kozhuharova, 2010). Its multifaceted mechanisms include nutrient enhancement, hormone modulation, and production of antimicrobial agents. Moreover, its spore-forming capability provides resilience against environmental challenges, making it a promising candidate for sustainable agriculture.

The exploration of endophytes in wild rice has revealed diverse species with growth promoting and antagonistic attributes. These findings underscore the significance of wild rice as a repository of valuable microbial resources that are essential for eco-friendly farming methods and the preservation of crop genetic diversity. *B. subtilis*, a versatile bacterium, has been demonstrated to be a growth promoter and pathogen controller (Ryu et al., 2003; Hayat et al., 2010; Fan et al., 2017). Its array of benefits includes eliciting systemic resistance and withstanding adverse conditions. *B. subtilis* strains exhibit a spectrum of effects, ranging from iron mobilization to ethylene modulation (Xie et al., 2014; Freitas et al., 2015; Woo et al., 2020). Variants such as GBO3, OKB105, and GOT9 show strain-specific advantages, ranging from iron migration to drought tolerance. Notably, *B. subtilis* JA has demonstrated the potential as a biocontrol agent *via* volatile inhibition (Chen et al., 2008), whereas *B. subtilis* SS12.9 displays iturin-based biocontrol against *Xanthomonas oryzae* pv. Oryzae (Beri et al., 2012).

The present study corroborated the antagonistic potential of the G5 strain against *M. oryzae* using multiple approaches, including SEM, plate confrontation, and biocontrol assays. The unique morphological alterations in *M. oryzae* hyphae induced by G5 treatment underscore its distinct mechanism of action (Sha et al., 2020; Chen et al., 2021). Disruption of appressorium formation emerged as a key facet of the effects of G5 on *M. oryzae*, in contrast to previous studies (Chen et al., 2019; Liu et al., 2021). In their study, antagonistic bacteria inhibited both conidial germination and appressorium formation of *M. oryzae*. This underscores the pivotal role of the appressorium in *M. oryzae* infection and highlights the potential for G5 to mitigate rice blast by impeding infection structure formation.

The G5 genome hosts intriguing antibiotic synthesis genes, such as surfactin, fengycin, and bacillaene, which are known for their antagonistic properties (Heerklotz and Seelig, 2007; Erega et al., 2021). These compounds confer broad-spectrum antibacterial and antifungal properties, suggesting application potential for G5. Additionally, the novel identified gene clusters indicate unexplored antibacterial substances. The G5 strain also contains genes related to plant growth promotion, such as those linked to spermidine synthesis and acetoin production (Zhang et al., 2015; Nascimento et al., 2019). These attributes indicate their potential to bolster plant development and systemic resistance, which warrant further investigation. The diverse biosynthetic abilities of *B. subtilis* warrant attention and customization for specific applications. It is essential to recognize the disparity between *B. subtilis* strains and optimize the fermentation conditions or conduct genetic modifications to maximize efficacy.

## 5. Conclusion

In summary, we successfully isolated the endophytic antagonistic bacterium G5 from *O. officinalis* Wall. and identified it as a strain of *B. subtilis* through morphological and genetic analyses. G5 exhibits potent biocontrol effects against *M. oryzae via* distinct mechanisms. Its genome holds promise for understanding pathogen inhibition and microbial inoculum development. Harnessing the potential of *B. subtilis* G5 will require tailored approaches and optimization to maximize its role in agriculture.

## Data availability statement

The data presented in this study are deposited in the National Center for Biotechnology Information (NCBI) GenBank (https://www.ncbi.nlm.nih.gov/genbank/) under accession number OQ874691. The assembled sequences were deposited in the NCBI (BioProject ID: PRJNA997266).

## Author contributions

L-YL: Writing—original draft. Z-XX: Data curation, Writing—original draft. J-LL: Conceptualization, Writing—original draft. D-ZY: Methodology, Writing—review and editing. LL: Investigation, Writing—review and editing. LC: Resources, Writing—review and editing. Q-FZ: Project administration, Writing—review and editing. F-YY: Formal analysis, Writing—review and editing. R-XL: Data curation, Writing—original draft. Z-QC: Conceptualization, Writing—review and editing. S-QX: Resources, Writing—review and editing.
