# Supplementary material for: Biological control of Magnaporthe oryzae using natively isolated Bacillus subtilis G5 from Oryza officinalis roots
Source: Front Microbiol. 2023 Oct 9;14:1264000. doi: 10.3389/fmicb.2023.1264000 (PMC10591090; doi:10.3389/fmicb.2023.1264000)
Supplement: Supplementary file 1 [file Data_Sheet_1.pdf]

Supplementary Material

1 Supplementary Figures

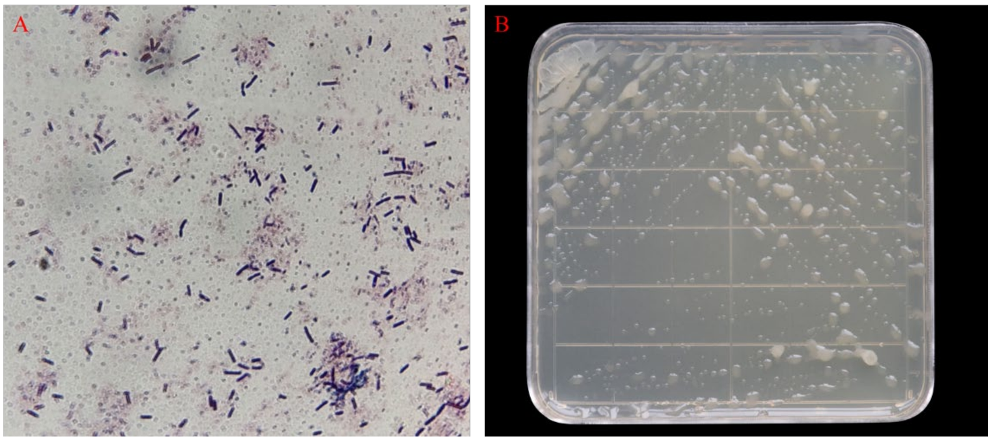

Supplementary Figure 1. Morphology of endophytic bacterium strain G5. A) Gram staining of G5. (B) Colony morphology of G5 grown for 24 h on LB agar plate.

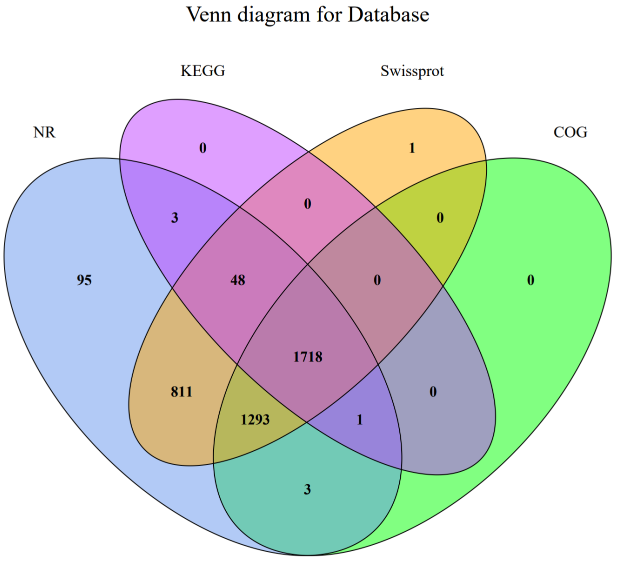

Supplementary Figure 2. Function annotation were performed by BlastX against the protein databases NR, SwissProt, COG and KEGG
